# Evaluating two decision aids for Australian men supporting informed decisions about prostate cancer screening: A randomised controlled trial

**DOI:** 10.1371/journal.pone.0227304

**Published:** 2020-01-15

**Authors:** Kristen Pickles, Luise Kazda, Alexandra Barratt, Kevin McGeechan, Jolyn Hersch, Kirsten McCaffery

**Affiliations:** Sydney School of Public Health, Faculty of Medicine and Health, The University of Sydney, Sydney, Australia; Centro per lo Studio e la Prevenzione Oncologica, ITALY

## Abstract

**Background:**

Australian clinicians are advised to ‘offer evidence-based decisional support to men considering whether or not to have a PSA test’. This randomised trial compared the performance and acceptability of two new decision aids (DAs) to aid men in making informed choices about PSA screening.

**Methods:**

~3000 Australian men 45–60 years with varying educational attainment were recruited via an online panel and randomised to view one of two online decision aids (one full length, one abbreviated) and completed a questionnaire. The primary outcome was informed choice about PSA screening.

**Findings:**

Significantly more men in the long DA group (38%) made an informed choice than men who received the shorter DA (33%) (95% CI 1.1% to 8.2%; p = 0.008). On knowledge, the long DA group scored, on average, 0.45 points higher than the short DA group (95% CI 0.14 to 0.76; p = 0.004) and 5% more of the participants achieved an adequate knowledge score (95% CI 1.9% to 8.8%; p = 0.002). Men allocated the long DA were less likely to intend to have a PSA test in the future (53%) than men in the short DA group (59%). Both DAs rated highly on acceptability.

**Conclusions:**

Both DAs were useful and acceptable to men regardless of education level and both supported informed decision making. The long version resulted in higher knowledge, and a higher proportion of men able to make an informed choice, but the differences were small. Long DAs may be useful for men whose informational needs are not satisfied by a short DA.

The balance between the benefits and harms of screening for prostate cancer remains controversial. There is no population screening programme for prostate cancer in Australia however Australia has high ad hoc screening rates [1,2] with patient request a common driver. [3,4] In 2017 the United States Preventive Services Task Force (USPSTF) assigned prostate cancer screening a “C” recommendation for men 55–69 years, concluding that the potential benefits and adverse impacts of prostate-specific antigen (PSA)-based screening are closely balanced in that age group.[5] Current international guidelines emphasise that the decision should be an individual one, based on personal values and preferences.[5–7]

In response, there is much focus on developing and disseminating health care information to assist men to make informed choices. Making an informed, evidence-based decision is especially important in screening of asymptomatic people ‘because there is no medical urgency for intervention or treatment and therefore choices are made in a preference-sensitive decision setting’. [8]

To enable a person to make an informed choice they need to be given adequate, high-quality, relevant, unbiased information on all possible consequences of the options. [9] Decision aids are evidence-based tools designed to support participation in decision making and improve the quality of people’s health care decisions.[10] Providing information within a decision aid can increase informed choice about breast cancer screening.[11]

Randomised trials have consistently shown that men who use decision aids are better informed and less conflicted in prostate screening decisions when compared to usual care.[12] A 2012 Cochrane review concluded, however: ‘little is known about the degree of detail that decision aids need in order to have positive effects on attributes of the decision or decision-making process’.[13] To our knowledge, no study has compared the performance of a full-length with an abbreviated decision aid for men deciding whether or not to have a PSA screening test.

A person’s health literacy and educational status can affect their ability to use health information and services, so developers of decision aids need to ensure that tools are accessible to groups with lower and higher literacy and education.[14,15] Another important consideration of this research is therefore to assess how acceptable and effective these interventions may be for men with low levels of education and health literacy.

This study had three research questions:

1. Which decision aid is better at supporting informed decision making about prostate cancer screening in a community sample–a long or short decision aid?
2. Do the two decision aids differ on cognitive and psychological variables or measures of acceptability?
3. What is the impact of educational background on the performance of the decision aids?

## Methods

In 2012, the Prostate Cancer Foundation of Australia (PCFA) partnered with Cancer Council Australia (CCA) to develop a clinical practice guideline for prostate cancer screening in Australia [http://wiki.cancer.org.au/australiawiki/index.php?oldid=134877]. To support implementation of the guideline, development of an evidence-based decision aid for men considering having a PSA test, compliant with international standards for best practice, was recommended. We developed two decision aids, one long and one short (essentially an abbreviated version of the long form) to meet this need and conducted the evaluation described here.

### Purpose

**Fig 1:**
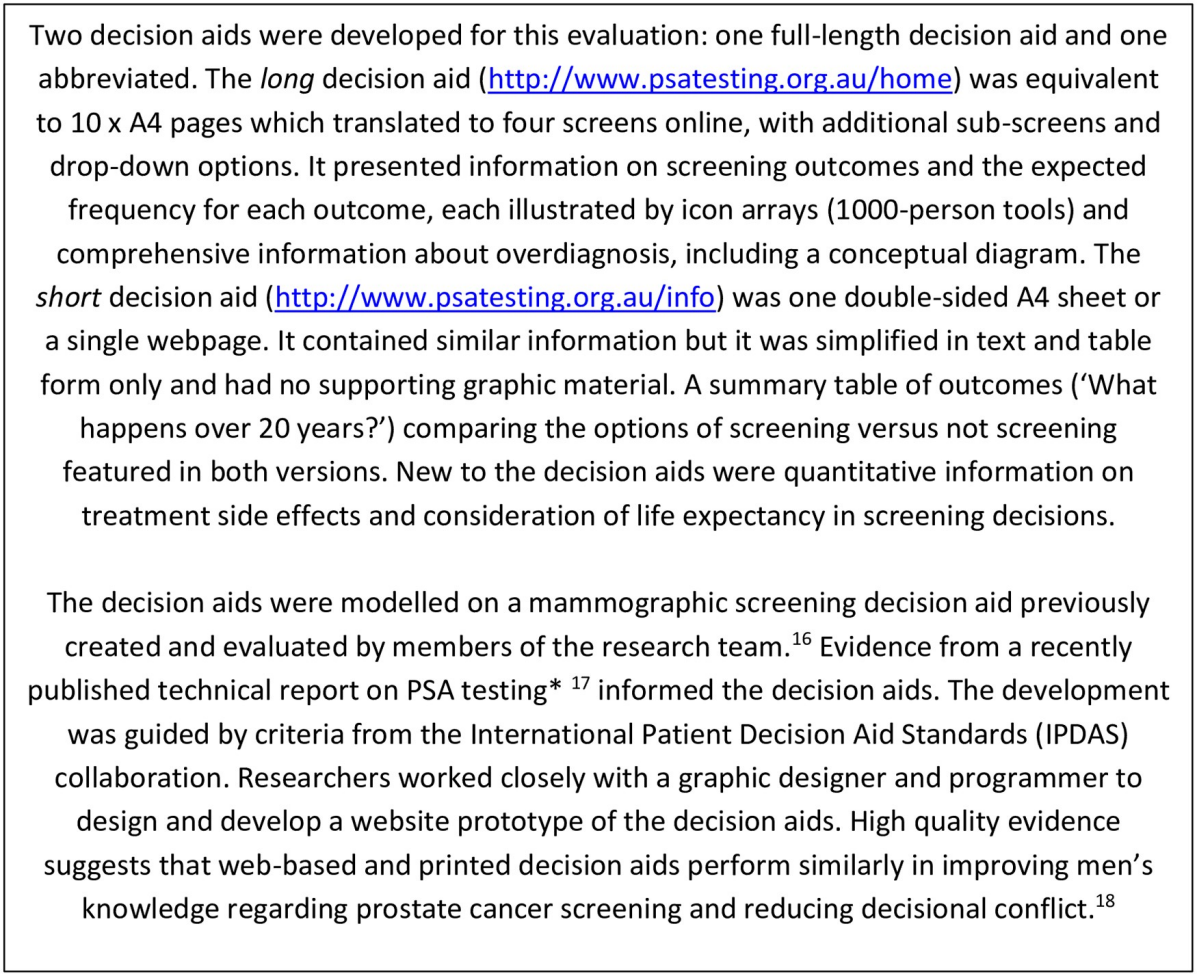
Description of the intervention and its development. *Estimates are based on 13 years of follow-up of men in the European Randomised Study of Prostate Cancer (ERSPC) and Australian data on PSA testing, prostate cancer incidence, prostate cancer mortality, and prostate cancer survival data to estimate cumulative risks of benefit and harm over 20 years of testing from ages 50 to 69 years. Full details of the estimation of these outcomes are given in S5 Appendix.[16–18].

To collect information from Australian men about the usefulness and acceptability of two decision aids, one long (10 pages) and one short (2 pages). See Fig 1.

### Design

Randomised controlled trial with participants randomised to view one of two online decision aids, either (a) a full-length decision aid, or (b) an abbreviated version of the decision aid. Participants completed a baseline questionnaire and answered further questions after viewing the online decision aid.

### Participants

Community sample of Australian males aged 45–60 years, recruited via an international survey sampling company frequently used in research studies. Quota sampling was used to ensure inclusion of men in relevant age groups and to obtain strong representation of men with lower educational attainment (i.e. school-level qualifications only). Eligibility criteria: (1) belonged to the survey sampling panel, (2) accepted an invitation to participate in the online questionnaire, (3) aged 45–60 years, and (4) did not have a prostate cancer diagnosis.

### Outcomes

The primary outcome was informed choice.[19] It comprises 3 constructs combining (1) adequate knowledge of possible outcomes of screening, and consistency between (2) a man’s attitude towards the screening test (positive or negative), and (3) intention to have a PSA test, to determine the proportion of men who made an informed (or uninformed) choice. We assessed both conceptual and numerical knowledge with a competency-based approach, [20] drawing on our team’s previous work.[16] Questions were asked to assess participants’ understanding of screening outcomes (mortality benefit, false positives, overdiagnosis) and awareness of the approximate numbers affected. Secondary outcomes were divided into two broad categories: (1) cognitive and psychological measures, including anticipated decisional regret, risk perceptions, and cancer worry, and (2) the use and acceptability of both decision aids. The acceptability of a DA refers to ratings regarding the comprehensibility of its components, its length, amount of information, balance in presentation of information about options, and overall suitability for decision making.

### Procedure

Outcomes were assessed via an online questionnaire that was developed using internationally accepted, validated scales and items in previous published studies that evaluated decision aids.[11,16,21] All men completed the same questionnaire. Standard socio-demographic data was obtained from participants and included personal history of cancer, family history of prostate cancer, and prostate cancer screening history.

### Analysis

Indicators of informed choice (knowledge, attitudes, intention) were scored according to a previously developed and tested marking scheme [11,16], amended minimally for our purposes (S1 Appendix). For published scales, responses were scored as per author coding instructions. For all analyses we compared either the proportion of men (categorical variables) or the mean (continuous variables) in the long and short decision aid arms. We stratified the primary outcome (informed choice) as well as some secondary outcomes (use and acceptability of DA) by education. For this purpose, we dichotomized highest educational attainment into lower (non-tertiary education) and higher levels (tertiary education).

A sample size of 3000 men (2000 45-54y, 1000 55-60y) was calculated to enable percentage estimates with confidence intervals of +/-4% (or less) within each randomised group, with stratification by education, and to enable us to detect a difference of approximately 7% between the randomised groups (in each stratum of education) in the proportion who found the aid acceptable and comprehensible, assuming acceptability proportions of 0.7 or more in each group. Our previous work with the development of a similar DA for breast cancer screening suggested the proportions finding the aids acceptable and understandable were likely to be in this range.[11] Analysis was by intention to treat. Categorical outcomes were analysed using a χ^2^ test, continuous outcomes with a two-sample t test (α of 5%, two-sided).

A sensitivity analysis was performed to account for missing data. We conducted multiple imputation by creating 20 imputed datasets using chained equations and pooling the resulting effect measures. With this process we imputed missing values for men who were randomised but did not answer all attitudes questions (n = 173, 5.8%). The sensitivity analysis provided similar results to the main analysis and conclusions were unchanged. Ethics approval was received from the Human Research Ethics Committee of the University of Sydney (2018/165). The trial was registered on the Australian New Zealand Clinical Trials Registry (ANZCTR: ACTRN12618001718235). All interested participants were directed to an online Participant Information Statement; subsequent completion and submission of the questionnaire was considered evidence of consent. Participants by virtue of being on the survey sampling database have already consented to being involved in online research.

## Results

**Fig 2:**
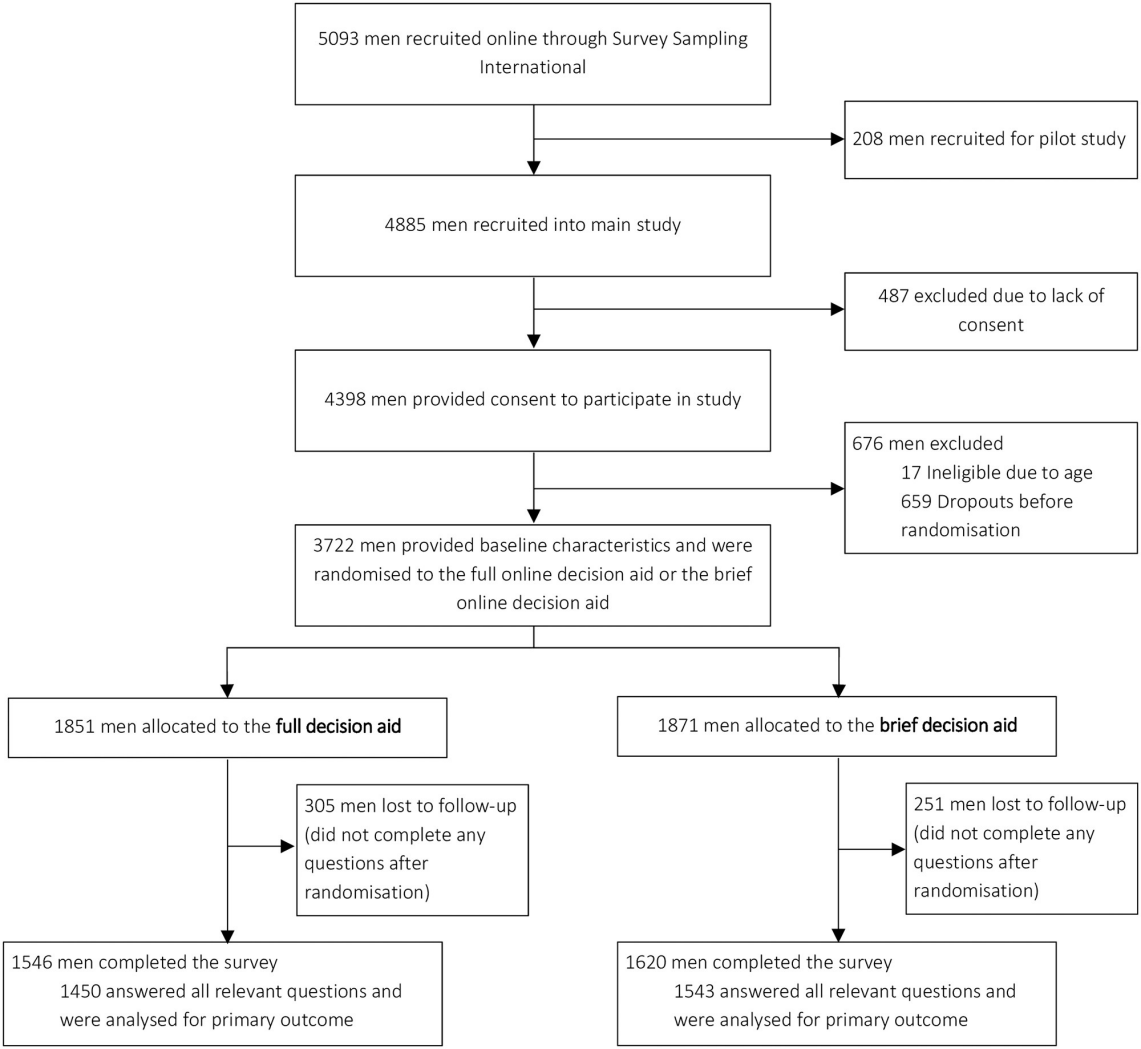
Study flowchart.

From 27 June to 26 July 2018, 5093 men aged 45 to 60 years were contacted by a panel survey sampling company, with 4885 men invited to the main study (208 men participated in a pilot study) (See Fig 2). A total of 4398 men consented; 676 were excluded because they did not meet the required age range or dropped out prior to randomisation. The remaining 3722 men were allocated at random to view one of the two DAs.

Of the 3722 participants (1851 allocated to the long DA and 1871 to the brief DA) a total of 2993 (80%) men completed all questions in the survey and were included in the analysis of the primary outcome. 173 men did not provide answers to all attitudes questions and were excluded from the analysis of the primary outcome but were included in relevant secondary analyses.

Baseline demographics were similar across both groups (Table 1). Overall, 55% of participants indicated that they had heard of the PSA test before and 38% of participants had previously had a PSA test (up to 51% in 55-60-year-old age group). Of these men, the majority (65%) stated that they had had a PSA test because their doctor had suggested it as part of a routine check-up. Around one-quarter of men who had received a PSA screening test indicated that their doctor ‘just conducted the blood test’ in a consultation.

**Table 1:** Baseline characteristics.

	Long DA (n = 1,546)	Brief DA (n = 1,620)
***Age***		
Median (IQR) age (years)^#^	52 (48–56)	52 (48–56)
***Education***		
No tertiary education	998 (65%)	1116 (69%)
Tertiary education	548 (35%)	504 (31%)
***Current Employment***		
Working full time	960 (62%)	983 (61%)
Working part time	185 (12%)	233 (14%)
No paid job	401 (26%)	404 (25%)
***Main language spoken at home***		
English	1476 (96%)	1542 (95%)
***Marital status***		
Married or living with partner	1066 (69%)	1111 (69%)
Widowed, divorced or separated	176 (11%)	196 (12%)
Single, never married	291 (19%)	301 (19%)
Prefer not to say	13 (1%)	12 (1%)
***Private Health Insurance***		
Yes	882 (57%)	912 (56%)
***Health Literacy***^		
Higher	1372 (89%)	1420 (88%)
Lower	174 (11%)	200 (12%)
***Family history of prostate cancer***		
No close blood relative ever diagnosed	1242 (80%)	1263 (78%)
At least one close blood relative ever diagnosed	304 (20%)	357 (22%)
***Past PSA experience***		
Ever heard of the PSA test	840 (54%)	895 (55%)
Ever had a PSA screening test	578 (37%)	624 (38%)
Ever had an abnormal PSA test	53 (3%)	78 (5%)
***Medical Maximizer-Minimizer Scale***^$^		
Overall mean score (S.D.)	4.46 (0.90)	4.49 (0.90)

^#^Data missing for 6 men in the long DA group and for 5 men in the short DA group

^Determined by asking how confident participants were in filling out medical forms by themselves on a scale of “extremely confident” (1) to “not at all confident” (5). Answers were dichotomised as “higher” including 1 and 2 and “lower” combining 3 to 5

^*$*^ Items were rated on a scale from “strongly disagree” (1) to “strongly agree” (7). The mean score for each respondent is recorded with a greater score indicating a preference towards seeking health care at a greater frequency than those scoring lower on the scale. This scale is based on Scherer et al [22]

### Which decision aid is better at supporting informed decision making about prostate cancer screening in a community sample—A long or short decision aid?

#### Primary outcome: Informed choice

A significantly higher proportion of men allocated the long DA (38%) were assessed to have made an informed choice about PSA screening than men who received the brief DA (33%) (4.7% more; 95% CI 1.1% to 8.2%; p = 0.008) (Table 2).

**Table 2:** Analysis of primary outcome.

	Long DA (n = 1,546)	Brief DA (n = 1,620)	Difference (95% CI)	p value
Informed choice*				
Made an informed choice	544/1450 (38%)	507/1543 (33%)	4.7 (1.2 to 8.1)	0.008
***Knowledge Score***^				
Mean (SD) total knowledge score	9.33 (4.52)	8.88 (4.30)	0.45 (0.14 to 0.76)	0.004
Adequate knowledge (≥9)	728 (47%)	676 (42%)	5.4 (1.9 to 8.8)	0.002
***Knowledge (numerical items)***^#^^@^				
How many men will still die from prostate cancer despite PSA testing				0.001^&^
Correct number	664 (43%)	593 (37%)	6.3 (2.9 to 9.7)	
Close to correct	106 (7%)	133 (8%)	-1.4 (-3.2 to 0.5)	
How many men will avoid dying from prostate cancer because of PSA testing				0.016^&^
Correct number	500 (32%)	462 (29%)	3.8 (0.6 to 7.0)	
Close to correct	137 (9%)	125 (8%)	1.1 (-0.8 to 3.1)	
How many men will be overdiagnosed with prostate cancer because of PSA testing				0.022^&^
Correct number	443 (29%)	536 (33%)	-4.4 (-7.6 to -1.2)	
Close to correct	183 (12%)	190 (12%)	0.1 (-2.1 to 2.4)	
***Knowledge (conceptual items)***^@^				
PSA screening will not find every prostate cancer	1128 (73%)	1113 (69%)	4.3 (1.1 to 7.4)	0.008
Not all men with an abnormal PSA test result have prostate cancer	1276 (83%)	1310 (81%)	1.7 (-1.0 to 4.4)	0.224
Men who do not have PSA screening tests are more likely to die from prostate cancer	1074 (70%)	1197 (74%)	-4.4 (-7.6 to -1.3)	0.006
Men who have PSA screening test are more likely to be diagnosed with prostate cancer	926 (60%)	949 (59%)	1.3 (-2.1 to 4.7)	0.451
Screening finds a cancer that would never have caused trouble is the best description for overdiagnosis	524 (34%)	398 (24%)	9.3 (6.2 to 12.5)	<0.001
Not all prostate cancers will eventually cause illness and death if they are not found and treated.	878 (57%)	791 (49%)	8.0 (4.5 to 11.4)	<0.001
When screening finds cancer, doctors cannot reliably predict whether it will cause harm.	683 (44%)	595 (37%)	7.5 (4.0 to 10.9)	<0.001
Screening leads some men with a harmless cancer to get treatment they do not need.	952 (62%)	886 (55%)	6.9 (3.5 to 10.3)	<0.001
Screening finds harmless cancers more often than it prevents death from prostate cancer.	748 (49%)	738 (46%)	2.8 (-0.6 to 6.3)	0.111
***Attitudes score***$				
Mean (SD) total attitudes score	5.71 (8.04)	6.78 (8.14)	-1.07 (-1.65 to -0.49)	<0.001
Positive score (>0)	1060/1450 (73%)	1179/1543 (76%)	-3.3 (-6.4 to -0.2)	0.037
Intentions about having a PSA screening test**				0.001^&^
Definitely will have screening	407 (26%)	488 (30%)	-3.8 (-6.9 to -0.7)	
Likely to have screening	410 (27%)	464 (29%)	-2.1 (-5.2 to 1.0)	
Unsure	478 (31%)	450 (28%)	3.1 (0.0 to 6.3)	
Not likely to have screening	201 (13%)	162 (10%)	3.0 (0.8 to 5.2)	
Definitely will not have screening	52 (3%)	56 (4%)	-0.1 (-1.4 to 1.2)	

*Informed choice defined as adequate knowledge and intentions consistent with attitudes (positive or negative)

^Total knowledge score was rated on a scale of 0 to 18 by adding up all conceptual and numeric knowledge questions. The threshold to determine “adequate knowledge” for informed choice was set a priori at more than 50% of total available knowledge marks, i.e. ≥9 points.

^#^2 points were given for a correct answer, 1 point was given for an answer deemed reasonably close to correct.

^@^Where data was missing for knowledge questions (conceptual and numeric knowledge) it was coded to “incorrect/don’t know”.

^$^Attitude items were rated from “strongly agree” (2) to “strongly disagree” (-2). Total scores could range from -24 to 24 with negative scores indicating a more negative attitude and positive scores indicating a more positive attitude. For informed choice, the threshold for a positive attitude was set at greater than zero. Data were missing for 173 participants (96 in the long DA group, 77 in the short DA group).

**This item was dichotomised as “positive intention to screen” (“definitely will” and “likely to”) and “negative intention to screen” (“unsure”, “not likely” and “definitely will not”) to estimate the “Informed choice” outcome.

^&^p value for difference in distribution of responses between groups.

A similarly small but significant difference in the two groups was observed in the knowledge score where participants in the long DA group scored, on average, 0.45 points higher than in the short DA group (95% CI 0.14 to 0.76; p value 0.004) and 5% more of the participants achieved an adequate knowledge score (95% CI 1.9% to 8.8%; p value 0.002). Men in the long DA group had significantly better understanding of the ‘best description for overdiagnosis’ (34%) than the short (24%) (9.3% difference; 95% CI 6.2% to 12.5%; p<0.001).

Attitudes were positive overall; participants in the short DA group reported a slightly more positive attitude towards PSA screening (1.07 points difference; 95% CI 0.49 to 1.65; p value <0.001). 6% more men from the short DA group (59%) intended to have a PSA test in the future compared with participants in the long DA group (53%) (95% CI 2.4% to 9.4%; p value 0.001). We further categorized men’s choices according to knowledge, attitudes, and intentions. 843 (28%) had inadequate knowledge but positive attitudes and intentions towards PSA screening. This was seen more frequently in men in the short (495/1543; 32%) than long group (348/1450; 24%) (p<0.001) (Table 3).

**Table 3:** Properties of screening choice.

	LONG DA (N = 1,450)^3^	BRIEF DA (N = 1,543)^3^	DIFFERENCE (95% CI)	P VALUE
***PROPERTIES OF SCREENING CHOICE***				<0.001^2^
**INFORMED CHOICE**^1^				
Made an informed choice to decline screening	189 (13%)	164 (11%)	2.4 (0.1–4.7)	
Made an informed choice to accept screening	355 (25%)	343 (22%)	2.3 (-0.8–5.3)	
**PARTLY UNINFORMED CHOICE**				
Made an inconsistent informed choice ^(Adequate knowledge bu itnconsistent attitudes and intentions to screen)^	181 (13%)	168 (11%)	1.6 (-0.7–3.9)	
Made a negative uninformed choice ^(Inadequate knowledge but consistent attitudes and intention to screen)^	140 (10%)	126 (8%)	1.5 (-0.6–3.5)	
**COMPLETELY UNINFORMED CHOICE**				
Made an uninformed choice to accept screening ^(Inadequate knowledge and inconsistent attitudes and intentions to screen)^	348 (24%)	495 (32%)	-8.1(-11.3–-4.9)	
Made an uninformed choice to decline screening ^(Inadequate knowledge and inconsistent attitudes and intentions to screen)^	237 (16%)	247 (16%)	0.3 (-2.3–3.0)	

^1^Defined as adequate knowledge and consistent attitudes and intentions (positive or negative)

^2^p value for difference in distribution of responses between groups

^3^ Data were missing for 173 participants (96 in the long DA group, 77 in the short DA group)

A sensitivity analysis for the primary outcome was conducted using alternative criteria to define adequate knowledge (i.e. defined adequate knowledge using conceptual items only), because there is little consensus in the literature regarding what level of knowledge constitutes being informed. In this sub-analysis men had to score at least four correct out of the six main conceptual items (S2 Appendix). With this knowledge threshold, an informed choice was made by 6662 men (46%) allocated to the long DA and 670 men (43%) who viewed the short (p = 0.219).

We also conducted a per protocol analysis including only those men who said they read all or most of the DA. 50% of men who read all or most of the short DA and 57% of men who read all or most of the long DA achieved adequate knowledge (7.4% more; 95% CI 3.5% to 11.4%; p value 0.001). 39% of men in the short and 45% in the long condition who read all or most of the information reached an informed choice [5.9 (2.0 to 9.9<0.003)].

### Do the two decision aids differ on cognitive and psychological variables and measures of acceptability?

#### Secondary outcomes: Cognitive and psychological variables

The groups differed on three items when distributions of the cognitive and psychological measures were compared: worry, anticipated regret, and perceived risk (Table 4). Men in the long DA group were less likely than men in the short DA group to feel that they might later regret not having a PSA screening test (p = 0.002). Men in the long DA group generally perceived their risk of developing prostate cancer as lower than men in the short DA group (p = 0.039). These differences were significant, but small.

**Table 4:** Analysis of secondary outcomes.

	Long DA (n = 1,546)	Brief DA (n = 1,620)	Difference (95% CI)	p value
Cancer worry*				
Worry about prostate cancer				0.010^&^
Not worried at all or a bit worried	1331/1453 (92%)	1370/1543 (89%)	2.8 (0.7 to 4.9)	
Quite worried or very worried	122/1453 (8%)	173/1543 (11%)	-2.8 (-4.9 to -0.7)	
Anticipated regret*				
Might later regret if do not screen				0.002^&^
(Strongly) agree	838/1453 (58%)	975/1543 (63%)	-5.5 (-9.0 to -2.0)	
Neither agree nor disagree	495/1453 (34%)	435/1543 (28%)	5.9 (2.6 o 9.2)	
(Strongly) disagree	120/1453 (8%)	133/1543 (9%)	-0.4 (-2.4 to 1.6)	
Might later regret if do screen				0.106^&^
(Strongly) agree	308/1453 (21%)	320/1543 (21%)	0.5 (-2.5 to 3.4)	
Neither agree nor disagree	535/1453 (37%)	519/1543 (34%)	3.2 (-0.2 to 6.6)	
(Strongly) disagree	610/1453 (42%)	704/1543 (46%)	-3.6 (-7.2 to -0.1)	
***Perceived risk***^#^				
Perceived risk of prostate cancer				0.039^&^
No chance or low chance	969/1452 (67%)	974/1543 (63%)	3.6 (0.2 to 7.0)	
Medium chance or high chance	483/1452 (33%)	569/1543 (37%)	-3.4 (-7.0 to 0.2)	
Perceived risk of prostate cancer relative to the average man				0.604^&^
Lower	471/1452 (32%)	484/1543 (31%)	1.1 (-2.3 to 4.4)	
About the same	808/1452 (56%)	858/1543 (56%)	0.0 (-3.5 to 3.6)	
Higher	173/1452 (12%)	201/1543 (13%)	-1.1 (-3.5 to 1.3)	

*Data were missing for 170 participants.

^#^Data were missing for 171 participants.

^&^p value for difference in distribution of responses between groups.

#### Secondary outcomes: Use and acceptability of decision aids

Table 5 shows how men used and evaluated the decision aids. Participants generally spent less time reading the short DA (p<0.001) and more men in the short DA group indicated that they had read most or all of it (3% difference, p<0.031). More men in the long DA group found the DA too long (14% difference, p<0.001).

**Table 5:** Use and acceptability of decision aids.

	Long DA (n = 1,546)	Brief DA (n = 1,620)	p value
***Perceived credibility (SD)***			
Information can be trusted	4.04 (0.93)	4.00 (0.94)	0.245
Information is accurate	3.96 (0.91)	3.91 (0.92)	0.091
Information is fair	4.02 (0.90)	3.96 (0.94)	0.072
Information tells the whole story	3.81 (0.99)	3.69 (0.99)	0.001
Information is unbiased	3.84 (0.98)	3.80 (0.97)	0.318
Total credibility score	3.93 (0.84)	3.87 (0.82)	0.037
***Time spent on reading the decision aid***			<0.001^&^
<5 minutes	502/1529 (33%)	946/1601 (59%)	
5–10 minutes	754/1529 (49%)	611/1601 (38%)	
10–20 minutes	255/1529 (17%)	40/1601 (3%)	
>20 minutes	18/1529 (1%)	4/1601 (0%)	
***Amount of decision aid read***			0.031^&^
All/most	1178/1529 (77%)	1284/1601 (80%)	
Some/little	351/1529 (23%)	317/1601 (20%)	
***Information in decision aid was new***			0.189^&^
None/some	798/1529 (52%)	798/1601 (50%)	
Most/all	731/1529 (48%)	803/1601 (50%)	
***Length of decision aid***			<0.001^&^
Much too short or a little too short	23/1529 (2%)	48/1601 (3%)	
Just about right	911/1529 (60%)	1157/1601 (72%)	
A little too long or much too long	595/1529 (39%)	396/1601 (25%)	
***Balance of decision aid***			0.482^&^
Clearly/a little slanted towards screening	497/1529 (33%)	525/1601 (33%)	
Completely balanced	786/1529 (51%)	843/1601 (53%)	
A little/clearly slanted away from screening	246/1529 (16%)	233/1601 (15%)	
***Decision aid was clear and easy to understand***			0.149^&^
Strongly agree or agree	1222/1529 (80%)	1322/1601 (83%)	
Neither agree nor disagree	259/1529 (17%)	239/1601 (15%)	
Strongly disagree or disagree	48/1529 (3%)	40/1601 (3%)	
***Found decision aid helpful in making decisions***			0.689^&^
Strongly agree or agree	1108/1529 (73%)	1182/1601 (74%)	
Neither agree nor disagree	364/1529 (24%)	363/1601 (23%)	
Strongly disagree or disagree	57/1529 (4%)	56/1601 (4%)	
***Would recommend decision aid to other men***			0.817^&^
Strongly agree or agree	1102/1529 (72%)	1145/1601 (72%)	
Neither agree nor disagree	366/1529 (24%)	385/1601 (24%)	
Strongly disagree or disagree	61/1529 (4%)	71/1601 (4%)	

^&^p value for difference in distribution of responses between groups.

Overall, participants in the longer DA group perceived it to be slightly more credible than those in the shorter DA group (0.06 points, p = 0.037). However, even though statistically significant, the actual difference was minimal. No significant differences were observed about how much of the information was new to participants, how balanced, clear and helpful they found the DA, or if they would recommend it to other men.

### What is the impact of educational background on the performance of the decision aids?

#### Outcomes stratified by education

Regression models showed no statistically significant evidence that the effect of the intervention differed by education level, with few exceptions (S3 and S4 Appendices). The only exceptions were “time spent on reading the decision aid” (p value = 0.016), and “information in decision aid was new” (p value = 0.040).

## Discussion

It is of utmost importance that men are given adequate information and a genuine opportunity to make an informed decision about whether or not to have a PSA test. In the present study, an abbreviated version of a decision aid increased knowledge and enabled informed choices about PSA screening almost as well as a full-length decision aid. The effect of the intervention was no different among men with lower or higher levels of education. These findings make an important contribution: few studies have compared a short and a long DA, and even fewer among men with a known, broad spread of educational attainment. A sub-analysis comparing detailed and simpler decision aids was included in a 2011 Cochrane review and similarly reported only a marginal improvement in knowledge for the detailed versions.[13] This is noteworthy because if a short DA presenting minimum, but enough, information can demonstrate effectiveness and rate favorably on measures of usefulness and acceptability as in this study, then this is likely to be universally preferred–at least in the first instance with options for further information available for those who desire it.

There are benefits to a shorter DA that works well enough and offers a solution to concerns about feasibility. We recognise and acknowledge some losses in terms of knowledge and in-depth understanding (it did appear that on the more difficult knowledge questions, participants in both educational strata performed better if they viewed the long version), but the short DA is practical and realistic and, importantly, does not disadvantage men with lower education.[14,15,21] Our study was conducted in Australia, where the clinical context and uptake of screening using the PSA test is similar to that in the USA and Canada.[23] Our findings are therefore applicable and relevant to those jurisdictions.

The percentage of men who achieved informed choice in this study is consistent with other DAs promoting informed choice internationally, including in the context of breast (15–24% of women)[11] and bowel screening (34%).[21] Although the absolute rate of adequate knowledge and informed choice overall seems low, we observed that more men reached adequate knowledge and were able to make an informed choice when they read the DA all the way through. We highlight that this was achieved with a single reading of the DA without support from any other source, e.g. a physician in a consultation.

Around 50% of men in both conditions indicated that they had seen the term ‘overdiagnosis’ before but, overall, understanding of the overdiagnosis information was particularly low, suggesting a need for targeted community education and engagement specifically around overdiagnosis.[24] Importantly, however, men exposed to the long version of the DA appeared to understand overdiagnosis better than men receiving the short form. This finding offers useful insight on methods to explain overdiagnosis, which is generally considered a difficult concept to convey. Identifying effective strategies to improve public knowledge about the downsides of cancer screening, particularly overdiagnosis, remains a priority for future research.

The effect of DAs on screening intentions and behaviour has not been consistent; in the prostate screening context, a recent Cochrane review reported that decision aids reduced the number of people choosing PSA screening when compared to usual care (RR 0.88; 95% CI 0.80–0.98; 10 studies; N = 3996). [10] In our study, the majority of men indicated positive attitudes toward screening after viewing the DA, slightly more in the short DA group, and around half of the men overall still intended to have a PSA test in the future.

The majority of participants interpreted the DA as neither recommending for nor against PSA screening, however a large minority believed the aid recommended screening. This is similar to other studies (e.g. [25]). Our findings that men remained positive and found the DA to be favoring screening highlight that it may take more than a single exposure to information like this for men to grasp the complexity of a message that is probably counter to their usual understanding and practices, including men who have had a PSA test previously.

### Strengths and limitations

We conducted a rigorous evaluation of the performance and acceptability of a short and long DA outside of the clinical setting with a large sample, including a large proportion of men with lower educational attainment. Comparing the impact among high and low education samples is rarely done and is important to ensure equity in SDM.[15]

The two DAs were evaluated by men registered with a survey sampling company who may or may not have been engaged and motivated by issues of PSA screening. It is therefore possible that our findings are an underestimate of the outcomes that would be achieved if the DAs were implemented in practice. It is likely that the information would have more salience for men receiving the DA in general practice or searching for it online, resulting in higher motivation to engage with the issues contained in the DAs.

## Conclusion

Both decision aids were useful and acceptable to men regardless of education level and both supported informed decision making. The long version resulted in small but significantly higher levels of knowledge, particularly around the unfamiliar topic of overdiagnosis. We suggest that the best approach at this time is to widely disseminate a printed version of the short decision aid for doctors to distribute in primary care, with the longer version made available online for those patients who, either then or later, may want to consider the question further. The longer online version could be promoted in the short-printed version and made available publicly as part of a broader strategy to disseminate information about the benefits and harms, including overdiagnosis and overtreatment, of screening for prostate cancer.

## Supporting information

S1 Appendix (DOCX)

S2 Appendix Conceptual knowledge items only scoring. The six conceptual items used in the sub-analysis were 22b, c, d, 24a, c and d.
(DOCX)

S3 Appendix Analysis of primary outcomes—By education. (DOCX)

S4 Appendix Analysis of secondary outcomes—Use & acceptability of decision aid by education. (DOCX)

S5 Appendix (DOCX)

S6 Appendix (DOCX)

S1 Questions (DOCX)
