# Supplementary material for: Evaluating two decision aids for Australian men supporting informed decisions about prostate cancer screening: A randomised controlled trial
Source: PLoS One. 2020 Jan 15;15(1):e0227304. doi: 10.1371/journal.pone.0227304 (PMC6961909; doi:10.1371/journal.pone.0227304)
Supplement: S1 Appendix — (DOCX) [file pone.0227304.s001.docx]

**S1 Appendix**

| **Topic** | **Conceptual Knowledge** | **Numeric Knowledge** | | **Total** |
| --- | --- | --- | --- | --- |
|  | Multiple choice items: marks for right answers | Marks for absolute values | Extra marks for relative values about right |  |
| **Benefit** | BC1 (Q22c) screened men are less likely to die of PC  2 marks if correct | BN1(Q25b) Reduction in risk of PC death if screened  2 marks: 1 (1-2); 1 mark: 3-6 | BX: Relationship of deaths to deaths avoided  1 mark: PN2≥PN1 | **8** |
|  | BC2 (Q22a) Screening will not find every PC  1 mark if correct | BN2(Q25a) Remaining risk of PC death if screened  2 marks: 4 (2-8); 1 mark: 9-12 |  |  |
| **False positives** | FC1 (Q22b) Not all men with abnormal results have PC  1 mark if correct | N/A | N/A | **1** |
| **Overdiagnosis** | OC1 (Q22d) Screened men more likely diagnosed  1 mark if correct | ON1(Q25c) Risk of ODx due to screening  2 marks: 25 (11-39); 1 mark: 7-10 or 40-58 | OX: Relationship of ODx to deaths avoided  1 mark: ON1>PN1 | **9** |
|  | OC2 (Q24a) Not all PCs would cause illness and death  1 mark if correct |  |  |  |
|  | OC3 (Q24b) Can’t predict whether PC will cause harm  1 mark if correct |  |  |  |
|  | OC5 (Q24c) Some get treatment they don’t need  1 mark if correct |  |  |  |
|  | OC6 (Q24d) Find harmless PC more than prevent death  1 mark if correct |  |  |  |
|  | OC7 (Q23) Overdiagnosis v false-positives distinction  1 mark if correct |  |  |  |
| **Total** | **10** | **6 marks** | **2 marks** | **18** |
