# Supplementary material for: Evaluating two decision aids for Australian men supporting informed decisions about prostate cancer screening: A randomised controlled trial
Source: PLoS One. 2020 Jan 15;15(1):e0227304. doi: 10.1371/journal.pone.0227304 (PMC6961909; doi:10.1371/journal.pone.0227304)
Supplement: S2 Appendix — The six conceptual items used in the sub-analysis were 22b, c, d, 24a, c and d. (DOCX) [file pone.0227304.s002.docx]

**S2 Appendix**

Conceptual knowledge items only scoring. The six conceptual items used in the sub-analysis were 22b, c, d, 24a, c and d.

|  | LONG DA (n=1,546) | Brief DA (n=1,620) | Difference (95% CI) | p value |
| --- | --- | --- | --- | --- |
| *Informed choice** |  |  |  |  |
| Made an informed choice | 662/1450 (46%) | 670/1543 (43%) | 2.2 (-1.3 to 5.8) | 0.219 |
| *Knowledge Score^* |  |  |  |  |
| Mean (SD) total knowledge score | 3.79 (1.73) | 3.62 (1.69) | 0.16 (0.43 to 0.28) | 0.008 |
| Adequate knowledge (>3) | 918 (59%) | 901 (56%) | 3.8 (0.3 to 7.2) | 0.032 |

^*^Informed choice defined as adequate knowledge and intentions consistent with attitudes (positive or negative)

^Total knowledge score was rated on a scale of 0 to 6 by adding up the main six conceptual knowledge items. The threshold for “adequate knowledge” was set at 4 out of 6 questions answered correctly.
