# Supplementary material for: Evaluating two decision aids for Australian men supporting informed decisions about prostate cancer screening: A randomised controlled trial
Source: PLoS One. 2020 Jan 15;15(1):e0227304. doi: 10.1371/journal.pone.0227304 (PMC6961909; doi:10.1371/journal.pone.0227304)
Supplement: S3 Appendix — (DOCX) [file pone.0227304.s003.docx]

**S3 Appendix**

**ANALYSIS OF PRIMARY OUTCOMES – By Education**

|  | Higher Education^$^ | | | Lower Education^$^ | | |  |
| --- | --- | --- | --- | --- | --- | --- | --- |
|  | **Long DA (n=916)** | **Brief DA (n=942)** | **p value** | **Long DA (n=630)** | **Brief DA (n=678)** | **p value** | **p value for interaction^$^** |
| *Informed choice** |  |  |  |  |  |  |  |
| Made an informed choice | 362/870 (42%) | 328/914 (36%) | 0.018 | 182/580 (31%) | 179/629 (28%) | 0.267 | 0.521 |
| *Knowledge Score^* |  |  |  |  |  |  |  |
| Mean (SD) total knowledge score | 10.00 (4.54) | 9.52 (4.38) | 0.020 | 8.35 (4.30) | 7.99 (4.03) | 0.119 | 0.694 |
| Adequate knowledge (>9) | 487 (53%) | 446 (47%) | 0.012 | 241 (38%) | 230 (34%) | 0.103 | 0.761 |

^*^Informed choice defined as adequate knowledge and intentions consistent with attitudes (positive or negative)

^Total knowledge score was rated on a scale of 0 to 18 by adding up all conceptual and numeric knowledge questions. The threshold for “adequate knowledge” was set at 50%, i.e. >9 points.

^$^interaction p values show that there is no effect modification by education level.
