# Supplementary material for: Evaluating two decision aids for Australian men supporting informed decisions about prostate cancer screening: A randomised controlled trial
Source: PLoS One. 2020 Jan 15;15(1):e0227304. doi: 10.1371/journal.pone.0227304 (PMC6961909; doi:10.1371/journal.pone.0227304)
Supplement: S4 Appendix — (DOCX) [file pone.0227304.s004.docx]

**S4 Appendix**

| Analysis of secondary outcomes – use & acceptability of decision aid by education | | | | | |
| --- | --- | --- | --- | --- | --- |
|  | **Higher Education^$^** | | **Lower Education^$^** | |  |
|  | **Long DA (n=916)** | **Brief DA (n=942)** | **Long DA (n=630)** | **Brief DA (n=678)** | **p value for interaction** |
| *Perceived credibility*(SD)* |  |  |  |  |  |
| Information can be trusted | 4.10 (0.91) | 4.07 (0.92) | 3.95 (0.96) | 3.90 (1.00) | 0.665 |
| Information is accurate | 4.03 (0.89) | 3.98 (0.90) | 3.87 (0.93) | 3.81 (0.93) | 0.770 |
| Information is fair | 4.06 (0.89) | 4.02 (0.92) | 3.97 (0.93) | 3.89 (0.97) | 0.619 |
| Information tells the whole story | 3.83 (0.99) | 3.71 (0.98) | 3.77 (0.99) | 3.65 (0.99) | 0.997 |
| Information is unbiased | 3.86 (0.98) | 3.86 (0.95) | 3.80 (0.97) | 3.73 (0.98) | 0.320 |
| Total credibility score | 3.97 (0.82) | 3.93 (0.80) | 3.87 (0.86) | 3.79 (0.84) | 0.612 |
| *Time spent on reading the DA^^^* |  |  |  |  | 0.016 |
| <5 minutes | 266/906 (29%) | 562/933 (60%) | 236/623 (38%) | 384/668 (58%) |  |
| 5-10 minutes | 468/906 (52%) | 345/933 (37%) | 286/623 (46%) | 266/668 (40%) |  |
| 10-20 minutes | *160/906 (18%)* | 24/933 (3%) | 95/623 (15%) | 16/668 (2%) |  |
| >20 minutes | 12/906 (1%) | 2/933 (0%) | 6/623 (1%) | 2/668 (0%) |  |
| *Amount of DA read* |  |  |  |  | 0.128 |
| All/most | 724/906 (80%) | 756/933 (81%) | 454/623 (73%) | 528/668 (79%) |  |
| Some/little | 182/906 (20%) | 177/933 (19%) | 169/623 (27%) | 140/668 (21%) |  |
| *Information in DA was new* |  |  |  |  | 0.040 |
| None/some | 505/906 (56%) | 470/933 (50%) | 293/623 (47%) | 328/668 (49%) |  |
| Most/all | 401/906 (44%) | 463/933 (50%) | 330/623 (53%) | 340/668 (51%) |  |
| *Term “overdiagnosis” seen before* |  |  |  |  |  |
| Yes | 513/906 (57%) | 538/933 (58%) | 295/623 (47%) | 289/668 (43%) | 0.366 |
| *Length of DA* |  |  |  |  | 0.771 |
| Much/a little too short | 13/906 (1%) | 31/933 (3%) | 10/623 (2%) | 17/668 (3%) |  |
| Just about right | 527/906 (58%) | 659/933 (71%) | 384/623 (62%) | 498/668 (75%) |  |
| A little/much too long | 366/906 (40%) | 243/933 (26%) | 229/623 (37%) | 153/668 (23%) |  |
| *Balance of DA* |  |  |  |  | 0.860 |
| Clearly/a little slanted towards screening | 296/906 (33%) | 315/933 (34%) | 201/623 (32%) | 210/668 (31%) |  |
| Completely balanced | 450/906 (50%) | 468/933 (50%) | 336/623 (54%) | 375/668 (56%) |  |
| A little/clearly slanted away from screening | 160/906 (18%) | 150/933 (16%) | 86/623 (14%) | 83/668 (12%) |  |
| *DA was clear and easy to understand* |  |  |  |  | 0.467 |
| Strongly agree/agree | 738/906 (82%0 | 788/933 (85%) | 484/623 (78%) | 534/668 (80%) |  |
| Neither agree nor disagree | 141/906 (16%) | 119/933 (13%) | 118/623 (19%) | 120/668 (18%) |  |
| Strongly disagree/disagree | 27/906 (3%) | 26/933 (3%) | 21/623 (3%) | 14/668 (2%) |  |
| *Found DA helpful in making decisions* |  |  |  |  | 0.959 |
| Strongly agree/agree | 667/906 (74%) | 703/933 (75%) | 441/623 (71%) | 479/668 (72%) |  |
| Neither agree nor disagree | 201/906 (22%) | 194/933 (21%) | 163/623 (26%) | 169/668 (25%) |  |
| Strongly disagree/disagree | 38/906 (4%) | 36/933 (4%) | 19/623 (3%) | 20/668 (3%) |  |
| *Would recommend DA to other men* |  |  |  |  | 0.751 |
| Strongly agree/agree | 667/906 (74%) | 672/933 (72%) | 45/623 (70%) | 473/668 (71%) |  |
| Neither agree nor disagree | 198/906 (22%) | 213/933 (23%) | 168/623 (27%) | 172/668 (26%0 |  |
| Strongly disagree/disagree | 41/906 (5%) | 48/933 (5%) | 20/623 (3%) | 23/668 (3%) |  |

^$^Higher Education comprised participants who indicated that their highest level of education was a university degree or a diploma or certificate. Lower education comprised participants who indicated that their highest level of education was a trade apprenticeship, higher school certificate or leaving certificate (or equivalent), school certificate or intermediate certificate (or equivalent) or had no school or other qualification.

^*^Perceived credibility items were measured on a scale of 1 to 5 with a higher total score indicating higher perceived credibility.

^^^19 values missing in the higher education group and 17 values missing in the lower education group.
