# Supplementary material for: Evaluating two decision aids for Australian men supporting informed decisions about prostate cancer screening: A randomised controlled trial
Source: PLoS One. 2020 Jan 15;15(1):e0227304. doi: 10.1371/journal.pone.0227304 (PMC6961909; doi:10.1371/journal.pone.0227304)
Supplement: S5 Appendix — (DOCX) [file pone.0227304.s005.docx]

**S5 Appendix**

Analysis was commissioned by the Prostate Cancer Foundation of Australia and undertaken by Associate Professor Mark Clements and supervised by Professor Bruce Armstrong. In the supplementary online material, Clements outlines the mathematical derivations for these estimates, lists the data used in the calculations, and provides R code used for the calculations.

<file:///C:/Users/kpic1741/AppData/Local/Microsoft/Windows/INetCache/Content.Outlook/CA8ID0G1/Draft%20web%20page%20170829.html>

This html document has been uploaded on the Prostate Cancer Foundation of Australia website and is referenced in the manuscript.

2. Clements M. Absolute risks of benefits and harms from PSA testing for use in the decision aid entitled "PSA testing for prostate cancer: It's your choice". June 2018
