# Supplementary material for: Evaluating two decision aids for Australian men supporting informed decisions about prostate cancer screening: A randomised controlled trial
Source: PLoS One. 2020 Jan 15;15(1):e0227304. doi: 10.1371/journal.pone.0227304 (PMC6961909; doi:10.1371/journal.pone.0227304)
Supplement: S6 Appendix — (DOCX) [file pone.0227304.s006.docx]

**S6 Appendix**

| An Online survey on  **Community evaluation of prostate cancer**  **screening decision support**  A study conducted by the School of Public Health at the University of Sydney  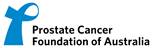  **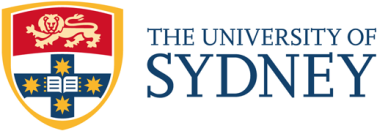** |
| --- |
