# Supplementary material for: Evaluating two decision aids for Australian men supporting informed decisions about prostate cancer screening: A randomised controlled trial
Source: PLoS One. 2020 Jan 15;15(1):e0227304. doi: 10.1371/journal.pone.0227304 (PMC6961909; doi:10.1371/journal.pone.0227304)
Supplement: S1 Questions — (DOCX) [file pone.0227304.s007.docx]

**SCREENING QUESTIONS**

**S1 Are you 45-60 years old?**

| **Yes** | **[CONTINUE]** |
| --- | --- |
| **No** | **[DISCONTINUE]** |

**S2 Have you ever been diagnosed with prostate cancer?**

| **Yes** | **[DISCONTINUE]** |
| --- | --- |
| **No** | **[CONTINUE]** |

**Would you like to receive a summary of the study results?**

| No |  |
| --- | --- |
| Yes, my email address is… |  |

**Is it okay for Sydney University researchers to contact you about participating in a telephone interview?**

| No |  |
| --- | --- |
| Yes, my name is… |  |
| Yes, my best contact number is… |  |

**Have any of your relatives been diagnosed with prostate cancer?**

| **Yes** |  |
| --- | --- |
| **No** |  |

**b. If yes, which of the following relatives have been diagnosed with prostate cancer?**

| Father |  |
| --- | --- |
| Son(s) |  |
| Brother(s) |  |
| Half-brother(s) |  |
| Nephew(s) |  |
| Uncle(s) |  |
| Grandfather(s) |  |

1. **Are you:**

| 45-54 years old |  |
| --- | --- |
| 55-60 years old |  |

1. **What is your age (in years)?**

|  |  |
| --- | --- |

1. **What state or territory do you live in?**

| New South Wales |  |
| --- | --- |
| Victoria |  |
| Queensland |  |
| Australian Capital Territory |  |
| Tasmania |  |
| South Australia |  |
| Northern Territory |  |
| Western Australia |  |

1. **Is the main language you speak at home English?**

| Yes |  |
| --- | --- |
| No |  |

1. **What is your highest level of education?**

| University degree |  |
| --- | --- |
| Diploma or certificate |  |
| Trade apprenticeship |  |
| Higher school certificate or leaving certificate (or equivalent) |  |
| School certificate or intermediate certificate (or equivalent) |  |
| No school or other qualifications |  |

1. **Which of the following categories best describes your current employment status?**

| Employed working full time |  |
| --- | --- |
| Employed working part-time |  |
| Not employed at the moment |  |
| Family caring / home duties |  |
| Retired |  |
| Studying full time |  |
| Prefer not to answer |  |

1. **What is your current relationship status?**

| Married or living with partner |  |
| --- | --- |
| Single, never married |  |
| Widowed |  |
| Divorced or separated |  |
| Prefer not to answer |  |

1. **Do you have private health insurance? (Private insurance also includes a Veteran’s gold card)**

| Yes |  |
| --- | --- |
| No |  |
| Don’t know |  |

1. **How confident are you filling out medical forms by yourself?**

| Extremely |  |
| --- | --- |
| Quite a bit |  |
| Somewhat |  |
| A little bit |  |
| Not at all |  |

1. **When making medical decisions (e.g. medication use, surgery), who is usually involved? Please tick as many boxes as relevant.**

| You |  |
| --- | --- |
| Your doctor |  |
| Your partner/spouse |  |
| Your adult child |  |
| Your friend |  |
| Your brother/s and/or sister/s |  |
| Another relative |  |
| A professional carer |  |
| Court appointed guardian |  |

1. **For each item below, please rate how much you personally agree or disagree with each statement.**
2. **It is important to treat disease even when it does not make a difference in survival**

| Strongly disagree |  |
| --- | --- |
| Disagree |  |
| Somewhat disagree |  |
| Neither agree nor disagree |  |
| Somewhat agree |  |
| Agree |  |
| Strongly agree |  |

1. **It is important to treat disease even when it does not make a difference in quality of life**

| Strongly disagree |  |
| --- | --- |
| Disagree |  |
| Somewhat disagree |  |
| Neither agree nor disagree |  |
| Somewhat agree |  |
| Agree |  |
| Strongly agree |  |

1. **Doing everything to fight illness is always the right choice**

| Strongly disagree |  |
| --- | --- |
| Disagree |  |
| Somewhat disagree |  |
| Neither agree nor disagree |  |
| Somewhat agree |  |
| Agree |  |
| Strongly agree |  |

1. **When it comes to health care, the only responsible thing to do is to actively seek medical care**

| Strongly disagree |  |
| --- | --- |
| Disagree |  |
| Somewhat disagree |  |
| Neither agree nor disagree |  |
| Somewhat agree |  |
| Agree |  |
| Strongly agree |  |

1. **If I have a health issue, my preference is to wait and see if the problem gets better on its own before going to the doctor**

| Strongly disagree |  |
| --- | --- |
| Disagree |  |
| Somewhat disagree |  |
| Neither agree nor disagree |  |
| Somewhat agree |  |
| Agree |  |
| Strongly agree |  |

1. **If I feel unhealthy, the first thing that I do is to go to the doctor and get a prescription**

| Strongly disagree |  |
| --- | --- |
| Disagree |  |
| Somewhat disagree |  |
| Neither agree nor disagree |  |
| Somewhat agree |  |
| Agree |  |
| Strongly agree |  |

1. **I often suggest that friends and family see their doctor**

| Strongly disagree |  |
| --- | --- |
| Disagree |  |
| Somewhat disagree |  |
| Neither agree nor disagree |  |
| Somewhat agree |  |
| Agree |  |
| Strongly agree |  |

1. **When it comes to health care, watching and waiting is never an acceptable option**

| Strongly disagree |  |
| --- | --- |
| Disagree |  |
| Somewhat disagree |  |
| Neither agree nor disagree |  |
| Somewhat agree |  |
| Agree |  |
| Strongly agree |  |

1. **If I have a medical problem, my preference is to go straight to a doctor and ask his or her opinion**

| Strongly disagree |  |
| --- | --- |
| Disagree |  |
| Somewhat disagree |  |
| Neither agree nor disagree |  |
| Somewhat agree |  |
| Agree |  |
| Strongly agree |  |

1. **When it comes to medical treatment, more is usually better**

| Strongly disagree |  |
| --- | --- |
| Disagree |  |
| Somewhat disagree |  |
| Neither agree nor disagree |  |
| Somewhat agree |  |
| Agree |  |
| Strongly agree |  |

1. **Have you heard of the prostate-specific antigen (PSA) test?**

| Yes |  |
| --- | --- |
| No |  |

1. **Have you ever had a prostate-specific antigen (PSA) test to screen for prostate cancer? (Screening behaviour)**

| No |  |
| --- | --- |
| Yes, I had a PSA test in the last 12 months |  |
| Yes, I had a PSA test 1-2 years ago |  |
| Yes, I had a PSA test more than 2 years ago |  |

1. **If yes, how did you come to have a PSA test? (more than one option may apply)**

| I asked about it |  |
| --- | --- |
| GP suggested as part of routine check-up |  |
| GP just conducted blood test |  |
| Wife/partner suggested it |  |
| Friend/relative suggested it |  |
| Experiencing urinary problems |  |
| Family history of prostate disease |  |

1. **If yes, have you ever had an abnormal prostate-specific antigen (PSA) test?**

| Yes |  |
| --- | --- |
| No |  |
| Don’t know |  |

*****VIEW DA HERE *****

1. **Think about the information you have just been given about screening for prostate cancer. Based only on what you have been told, please indicate how you feel about the information you were shown.**

|  | 1 | 2 | 3 | 4 | 5 |  |
| --- | --- | --- | --- | --- | --- | --- |
| Can’t be trusted | 🞏 | 🞏 | 🞏 | 🞏 | 🞏 | Can be trusted |
| Is inaccurate | 🞏 | 🞏 | 🞏 | 🞏 | 🞏 | Is accurate |
| Is unfair | 🞏 | 🞏 | 🞏 | 🞏 | 🞏 | Is fair |
| Doesn’t tell the whole story | 🞏 | 🞏 | 🞏 | 🞏 | 🞏 | Tells the whole story |
| Is biased | 🞏 | 🞏 | 🞏 | 🞏 | 🞏 | Is unbiased |

1. **At the moment, which of the following best describes your intentions about having a PSA screening test within the next 2-3 years?**

| You definitely will have a PSA test |  |
| --- | --- |
| You are likely to have a PSA test |  |
| You are unsure |  |
| You are not likely to have a PSA test |  |
| You definitely will not have a PSA test |  |

1. **The next few questions ask about your experience of using the decision aid:**
2. **About how long did you spend reading the decision aid?**

| <5 minutes |  |
| --- | --- |
| 5-10 minutes |  |
| 10-20 minutes |  |
| >20 minutes |  |

1. **How much of the decision aid did you read?**

| All the way through |  |
| --- | --- |
| Most |  |
| Some |  |
| A little |  |

1. **How much of the information was new to you?**

| None |  |
| --- | --- |
| Some |  |
| Most |  |
| All |  |

1. **Have you seen or heard the term ‘over-diagnosis’ before today?**

| Yes |  |
| --- | --- |
| No |  |
| Don’t know |  |

1. **How would you rate the length of the decision aid?**

| Much too long |  |
| --- | --- |
| A little too long |  |
| Just about right |  |
| A little too short |  |
| Much too short |  |

1. **How balanced did you find the information?**

| Clearly slanted towards screening |  |
| --- | --- |
| A little slanted towards screening |  |
| Completely balanced |  |
| A little slanted away from screening |  |
| Clearly slanted away from screening |  |

1. **You found the information clear and easy to understand**

| Strongly agree |  |
| --- | --- |
| Agree |  |
| Neither agree nor disagree |  |
| Disagree |  |
| Strongly disagree |  |

1. **You would find the decision aid helpful in making a decision about having a PSA test**

| Strongly agree |  |
| --- | --- |
| Agree |  |
| Neither agree nor disagree |  |
| Disagree |  |
| Strongly disagree |  |

1. **You would recommend this decision aid to other men thinking about having a PSA screening test**

| Strongly agree |  |
| --- | --- |
| Agree |  |
| Neither agree nor disagree |  |
| Disagree |  |
| Strongly disagree |  |

1. What other factors would influence your decision whether or not to have a PSA screening test? Please list as many as you wish.

| Free response |
| --- |
|  |
|  |
|  |
|  |

1. Do you have any further questions or comments about the decision aid?

(e.g. about using it, the information presented, what you think the main message of the decision aid is, what you liked/disliked about the aid). Do you have any suggestions for how it could be improved?

| Free response |
| --- |
|  |
|  |
|  |
|  |

1. The next few questions relate to information about prostate cancer screening using the PSA test**. You may return to the decision aid at any time. If you’re not sure of the answer, give it your best guess. The questions are not to test you, but rather to see whether information for men is clear and understandable.**
2. **Do you think a PSA screening test will find every prostate cancer?**

| Yes |  |
| --- | --- |
| No |  |
| *I really don’t know how to answer this question* |  |

1. **Do all men with an abnormal PSA test result have prostate cancer?**

| Yes |  |
| --- | --- |
| No |  |
| *I really don’t know how to answer this question* |  |

1. **Who do you think is more likely to die from prostate cancer?**

| Men who have PSA screening tests |  |
| --- | --- |
| Men who do not have PSA screening tests |  |
| *I really don’t know how to answer this question* |  |

1. **Who do you think is more likely to be diagnosed with prostate cancer?**

| Men who have PSA screening tests |  |
| --- | --- |
| Men who do not have PSA screening tests |  |
| *I really don’t know how to answer this question* |  |

1. **Which of these 2 statements best describes over-diagnosis?**

| Screening finds a cancer that would never have caused trouble |  |
| --- | --- |
| Screening finds an abnormality but extra tests show it is not cancer |  |
| *I really don’t know how to answer this question* |  |

1. Below are some statements about prostate cancer and screening. Please indicate whether you think each statement is TRUE or FALSE.

**If you’re not sure of the answer, give it your best guess.**

1. **All prostate cancers will eventually cause illness and death if they are not found and treated.**

| True |  |
| --- | --- |
| False |  |
| *I really don’t know how to answer this question* |  |

1. **When screening finds cancer, doctors can reliably predict whether it will cause harm.**

| True |  |
| --- | --- |
| False |  |
| *I really don’t know how to answer this question* |  |

1. **Screening leads some men with a harmless cancer to get treatment they do not need.**

| True |  |
| --- | --- |
| False |  |
| *I really don’t know how to answer this question* |  |

1. **Screening finds harmless cancers more often than it prevents death from prostate cancer.**

| True |  |
| --- | --- |
| False |  |
| *I really don’t know how to answer this question* |  |

1. **Remember, you may return to the decision aid at any time. If you’re not sure of the answer, give it your best guess.**

For the next three questions, **imagine 1000 men who have PSA testing every 2 years from 50 to 69 years.**

1. Out of these 1000 men, over 20 years, about how many will **still die from prostate cancer** despite PSA testing?
2. Out of these 1000 men, over 20 years, about how many will **avoid dying from prostate cancer** because of PSA testing?
3. Out of these 1000 men, over 20 years, about how many will be diagnosed, monitored, or treated for a prostate cancer that would not have caused any trouble (**over-diagnosis**)?
4. **How worried are you about developing prostate cancer?**

| Not worried at all |  |
| --- | --- |
| A bit worried |  |
| Quite worried |  |
| Very worried |  |

1. **Please indicate how strongly you agree or disagree with the following statements:**

**If you do NOT have a PSA screening test in the next few years, you may later wish you DID.**

| Strongly agree |  |
| --- | --- |
| Agree |  |
| Neither agree nor disagree |  |
| Disagree |  |
| Strongly disagree |  |

**If you DO have a PSA screening test in the next few years, you may later wish you did NOT.**

| Strongly agree |  |
| --- | --- |
| Agree |  |
| Neither agree nor disagree |  |
| Disagree |  |
| Strongly disagree |  |

1. **The next two questions ask how you feel about your risk of prostate cancer:**

**How likely do you think it is that you will develop prostate cancer in your lifetime?**

| No chance |  |
| --- | --- |
| Low chance |  |
| Medium chance |  |
| High chance |  |

**Compared with the average man your age, how would you rate your chances of developing prostate cancer sometime in your life?**

| Much lower |  |
| --- | --- |
| A bit lower |  |
| About the same |  |
| A bit higher |  |
| Much higher |  |

1. **The next few questions ask about your feelings towards prostate cancer screening, diagnosis, and treatment:**
2. **I believe treatment will always help men live longer**

| Strongly agree |  |
| --- | --- |
| Agree |  |
| Neither agree nor disagree |  |
| Disagree |  |
| Strongly disagree |  |

1. **If I had prostate cancer, I would prefer not to know**

| Strongly agree |  |
| --- | --- |
| Agree |  |
| Neither agree nor disagree |  |
| Disagree |  |
| Strongly disagree |  |

1. **Having the PSA test would reassure me**

| Strongly agree |  |
| --- | --- |
| Agree |  |
| Neither agree nor disagree |  |
| Disagree |  |
| Strongly disagree |  |

1. **Having a PSA test would do me more harm than good**

| Strongly agree |  |
| --- | --- |
| Agree |  |
| Neither agree nor disagree |  |
| Disagree |  |
| Strongly disagree |  |

1. **Even though a prostate biopsy can miss cancer, I would still have one**

| Strongly agree |  |
| --- | --- |
| Agree |  |
| Neither agree nor disagree |  |
| Disagree |  |
| Strongly disagree |  |

1. **If I had prostate cancer I believe I may live a better life without treatment**

| Strongly agree |  |
| --- | --- |
| Agree |  |
| Neither agree nor disagree |  |
| Disagree |  |
| Strongly disagree |  |

1. **If I have a PSA test I believe it could lead to treatment that is not necessary**

| Strongly agree |  |
| --- | --- |
| Agree |  |
| Neither agree nor disagree |  |
| Disagree |  |
| Strongly disagree |  |

1. **Having a PSA test would give me peace of mind**

| Strongly agree |  |
| --- | --- |
| Agree |  |
| Neither agree nor disagree |  |
| Disagree |  |
| Strongly disagree |  |

1. **Having a PSA test would cause me to worry unnecessarily**

| Strongly agree |  |
| --- | --- |
| Agree |  |
| Neither agree nor disagree |  |
| Disagree |  |
| Strongly disagree |  |

1. **I think the benefits of treatment for prostate cancer are more important than the side effects**

| Strongly agree |  |
| --- | --- |
| Agree |  |
| Neither agree nor disagree |  |
| Disagree |  |
| Strongly disagree |  |

1. **I think all men my age should have a PSA test for prostate cancer**

| Strongly agree |  |
| --- | --- |
| Agree |  |
| Neither agree nor disagree |  |
| Disagree |  |
| Strongly disagree |  |

1. **For me, having the PSA test is asking for trouble**

| Strongly agree |  |
| --- | --- |
| Agree |  |
| Neither agree nor disagree |  |
| Disagree |  |
| Strongly disagree |  |

1. **Have you ever been diagnosed with cancer?**

| Yes |  |
| --- | --- |
| No |  |
| Don’t know |  |

**If yes, which type of cancer? Please tick one or more answers.**

| Bowel |  |
| --- | --- |
| Breast |  |
| Lung |  |
| Lymphoma |  |
| Melanoma |  |
| Thyroid |  |
| Other |  |

1. **Has anyone in your immediate family (parents, siblings, children) ever been diagnosed with cancer?**

| Yes |  |
| --- | --- |
| No |  |
| Don’t know |  |

**If yes, which type of cancer? Please tick one or more answers.**

| Bowel |  |
| --- | --- |
| Breast |  |
| Cervical |  |
| Lung |  |
| Lymphoma |  |
| Melanoma |  |
| Prostate |  |
| Thyroid |  |
| Other |  |

1. **Would you say that your general health is:**

| Excellent |  |
| --- | --- |
| Very good |  |
| Good |  |
| Fair |  |
| Poor |  |

**That is the end of the questions, thank you for your participation in this survey.**
